# Evaluation of the potential for QTc prolongation with avelumab

**DOI:** 10.1007/s00280-019-03925-z

**Published:** 2019-09-03

**Authors:** Yulia Vugmeyster, Gülseren Güzel, Meliessa Hennessy, Anja H. Loos, Haiqing Dai

**Affiliations:** 1 Clinical Pharmacology, EMD Serono Research and Development Institute, Inc, 45 Middlesex Turnpike, Billerica, MA 01821 USA; 2 Global Clinical Development Immuno-Oncology, Merck KGaA, Darmstadt, Germany; 3 Global Clinical Development, EMD Serono Research and Development Institute, Inc, Billerica, MA USA; 4 Global Biostatistics, Merck KGaA, Darmstadt, Germany

**Keywords:** QT interval, QTc, Avelumab, PD-L1, Solid tumor, Cancer

## Abstract

**Purpose:**

To report integrated electrocardiogram (ECG) summary and exposure–QTc analyses for avelumab, a human immunoglobulin G1 monoclonal antibody that binds programmed cell death 1 ligand 1, to assess potential effects on cardiac repolarization.

**Methods:**

Data were pooled from three-phase 1/2 studies of patients with advanced solid tumors who received avelumab monotherapy (22,000 ECGs from 1818 patients). All analyses used 12-lead singlet ECGs taken using local ECG machines before and approximately 2 h after avelumab infusion on prespecified days. The exposure–QTc and outlier analyses used locally read ECGs; since larger variability is known to be associated with local reading, outlier ECGs were subsequently reevaluated by central read. QTc derived from Fridericia’s formula (QTcF) and a project-specific formula (QTcP) were analyzed. Multivariable linear mixed-effects models were used to describe the relationship between serum concentration of avelumab and QTc absolute value or change from baseline (ΔQTc).

**Results:**

Exposure–QTc models showed that the effect of avelumab on QTc or ΔQTc was minimal and not statistically significant for both QTcP and QTcF. In addition, models including avelumab concentration and diphenhydramine premedication use did not show a clinically meaningful effect on the QT interval. The frequency of QTc outliers in both short and long ranges was overestimated by local reads. Six patients (0.3%) were QTc outliers; all had either received concomitant medication known to cause QT prolongation or had a preexisting cardiac condition.

**Conclusion:**

Avelumab does not have any clinically relevant effect on cardiac repolarization.

## Introduction

Tumor cells exploit immune checkpoint mechanisms, such as the interaction between programmed cell death 1 protein (PD-1) and its ligand programmed cell death 1 ligand (PD-L1), to evade immune responses [1]. Expression of PD-L1 on tumor cells, which binds to PD-1 expressed on CD8^+^ T cells, inhibits T-cell-mediated destruction of tumor cells [2]. In recent years, several antibodies that inhibit PD-1 or PD-L1 have been approved as anticancer therapeutics [3].

Avelumab is a human immunoglobulin G1 monoclonal antibody that binds specifically to PD-L1, inhibiting the interaction with PD-1 and preventing T-cell exhaustion [2, 4]. Avelumab is approved in multiple countries worldwide for the treatment of metastatic Merkel cell carcinoma and locally advanced or metastatic urothelial carcinoma after disease progression with platinum-containing chemotherapy, and has recently been approved by the US Food and Drug Administration in combination with axitinib for the treatment of advanced renal cell carcinoma [5, 6]. Currently, numerous clinical trials of avelumab are ongoing in a range of tumor types [7–9].

During a clinical trial program, it is critical to determine whether a drug causes any harmful cardiac effects, such as arrhythmia [10]. Furthermore, prolongation of the QT interval (due to delayed cardiac repolarization) is associated with potentially fatal arrhythmias, including torsades de pointes [10, 11]. Drug-induced QT prolongation has been associated with inhibition of the potassium ion channel encoded by the *hERG* gene; the proposed mechanisms for this inhibition include disruption of hERG plasma membrane protein trafficking or blockage of the ion-channel cavity [12, 13]. Monoclonal antibodies, such as avelumab, are considered unlikely to cause QT prolongation because they are too large to cross plasma membranes and, as such, are unable to block the inner cavity of the hERG channel [14]; however, some monoclonal antibodies have been found to prolong the QT interval, the mechanism of which is still unknown [15]. Furthermore, cases of immune checkpoint inhibitor-related cardiotoxicity have been reported, which may have been associated with QT prolongation [16], and animal studies have shown that deficiency of CTLA-4 and PD-1 can cause autoimmune myocarditis [17]. Several small-molecule anticancer drugs have also been found to induce QT prolongation, including tyrosine kinase inhibitors, histone deacetylase inhibitors, and BRAF inhibitors [18].

For all drugs in clinical development, a thorough QT/corrected QT (QTc) analysis of the potential effect on cardiac repolarization is recommended by the International Conference on Harmonisation (ICH) of Technical Requirements for Registration of Pharmaceuticals for Human Use E14 [10]. In 2015, the ICH updated its guidance to recommend that a concentration–QTc-modeling approach, which uses a prespecified linear mixed-effects model, be used as a potentially alternative method to determine whether a drug has any clinically relevant effect on the QTc interval [19].

This manuscript presents integrated electrocardiogram (ECG) summary and exposure–QTc analyses, based on the concentration–QTc-modeling approach in accordance with the ICH, to assess the potential effect of avelumab on cardiac repolarization in patients with metastatic or locally advanced solid tumors who were enrolled in three separate clinical studies. In each study, patients received avelumab monotherapy. Premedication with acetaminophen and an antihistamine for the prophylaxis of infusion-related reactions was also required; therefore, many patients received diphenhydramine, and the effect of this premedication on QT prolongation was also included in the exposure–QTc modeling.

## Materials and methods

### Study design and treatment

This analysis pooled data from patients enrolled in three studies of avelumab monotherapy: JAVELIN Solid Tumor (NCT01772004), JAVELIN Solid Tumor JPN (NCT01943461), and JAVELIN Merkel 200 (NCT02155647).

JAVELIN Solid Tumor is a phase 1 study performed in patients with metastatic or locally advanced solid tumors, which included an initial dose-escalation phase [patients (*n* = 53) received avelumab doses of 1, 3, 10, or 20 mg/kg every 2 weeks (Q2W)] followed by a dose-expansion phase [all patients (*n* = 1650, as of June 9, 2016) received avelumab 10 mg/kg Q2W] [4, 7, 20]. JAVELIN Solid Tumor JPN is a phase 1 study performed in Japanese patients, comprising a dose-escalation phase in patients with metastatic or locally advanced solid tumors [patients (*n* = 17) received avelumab doses of 3, 10, or 20 mg/kg Q2W] and a dose-expansion phase in patients with gastric or gastroesophageal junction cancer [all patients (*n* = 34) received avelumab 10 mg/kg Q2W] [21]. JAVELIN Merkel 200 is a phase 2 study of avelumab 10 mg/kg Q2W in patients with metastatic Merkel cell carcinoma; this analysis included a cohort of patients (*n *= 88) who had received prior chemotherapy [22, 23].

Full eligibility criteria for each study have been reported previously [4, 7, 21, 22]. In all three studies, patients with clinically significant (i.e., active) cardiovascular disease, specifically cerebrovascular accident/stroke or myocardial infarction < 6 months prior to enrollment, unstable angina, congestive heart failure (New York Heart Association class ≥ II), or serious uncontrolled cardiac arrhythmia requiring medication, were excluded from enrollment. Patients received avelumab treatment until disease progression, unacceptable toxicity, or any protocol-specified criterion for withdrawal occurred. Prior to avelumab, most patients received premedication with an antihistamine, such as diphenhydramine (25–50 mg, modified per local standards), 30–60 min before each avelumab infusion (approximately 66–80% of all patients who received 10 mg/kg avelumab across the three studies also received diphenhydramine). All studies were conducted in accordance with the Good Clinical Practice guidelines as defined by the ICH and the Declaration of Helsinki, and all patients provided informed consent before starting treatment.

### QT analyses overview

A total of 22,000 ECGs from 1818 patients from the three studies were analyzed in the integrated ECG summary analyses. To identify mean changes in QTc absolute values and changes from baseline (ΔQTc) over the treatment period, descriptive statistics were used to analyze locally read ECGs. These locally read ECGs were also analyzed for potentially clinically significant abnormalities to determine outliers in QTc data at both short and long ranges. ECGs that displayed any of the following criteria were classified as “outliers”: heart rate ≤ 50 bpm and decrease from baseline ≥ 20 bpm; heart rate ≥ 120 bpm and increase from baseline ≥ 20 bpm; PR interval ≥ 220 ms and increase from baseline ≥ 20 ms; QRS interval ≥ 120 ms; QTcF or QTcP absolute interval values > 450 to ≤ 480 ms, > 480 to ≤ 500 ms, or > 500 ms; or ΔQTcF or ΔQTcP increase > 30 to ≤ 60 ms or > 60 ms.

Since it has been previously reported that larger variability is associated with local reading of ECGs compared with centralized reading [24], outlier ECGs were subsequently reevaluated by central read. In two further analyses, to compare the outliers from locally and centrally read ECGs, patients were re-sampled and their ECGs were reevaluated by central read; one analysis included a random sampling of patients with two available ECG measurements (one taken at baseline and one at an on-treatment timepoint thereafter) from JAVELIN Solid Tumor and JAVELIN Merkel 200 (*n* = 180 patients; 360 total ECGs were planned to be analyzed; however, only 264 were available for reevaluation by central read), while the second sample analysis included all patients in the dose-escalation cohorts of JAVELIN Solid Tumor (483 total ECGs taken at screening or baseline or during treatment were analyzed from 53 patients). For the ECG summary analyses, the data cutoff date for JAVELIN Solid Tumor and JAVELIN Merkel 200 was June 9, 2016; for JAVELIN Solid Tumor JPN, the cutoff was November 20, 2015.

To investigate any potential relationship between avelumab concentration and the QTc interval, all patients from the three studies with ≥ 1 matched pair of pharmacokinetic (PK) and ECG measurements prior to avelumab infusion and within 0–2 h after infusion (2119 paired observations from 670 patients from all three studies) were included in an exposure–QT analysis. For the exposure–QT analysis, the data cutoff date for JAVELIN Solid Tumor and JAVELIN Solid Tumor JPN was November 20, 2015; for JAVELIN Merkel 200, the cutoff was January 20, 2016, and March 3, 2016, for PK and ECG data, respectively.

### ECG assessments and summary analyses

12-Lead singlet ECG measurements were taken using a local ECG machine before each avelumab infusion and approximately 2 h after infusion Q2W until week 13 and then every 6 weeks (JAVELIN Solid Tumor and JAVELIN Merkel 200) or 4 weeks (JAVELIN Solid Tumor JPN) thereafter. Patients included in the ECG summary analyses had received ≥ 1 dose of avelumab and had undergone ≥ 1 ECG measurement during the on-treatment period (from day 1 of treatment until ≤ 30 days after last treatment or until subsequent treatment, whichever occurred first).

The QT interval was corrected to reduce the effect of heart rate using either Fridericiaʼs formula (${\text{QTcF}} = {\text{QT}}\sqrt[3]{\text{RR}}$; where RR = 60/heart rate) or a project-specific formula (${\text{QTcP}} = {\text{QT}} + \hat{b}*\left[ {1 - {\text{RR}}} \right]$; where RR = 60/heart rate and $\hat{b}$ is estimated from a linear regression ${\text{QT}} = a + \hat{b}*{\text{RR}}$), which was derived using all pooled baseline QT and RR data available.

Measurement of change (Δ) in QTcF/QTcP from baseline required both an evaluable on-treatment and baseline ECG read. ECGs within the treatment period were analyzed for trends using descriptive statistics of absolute values [95% confidence intervals (CIs)] and changes from baseline (90% CIs) for scheduled visits and by study and by dose. In addition, all ECGs were analyzed for potentially clinically significant abnormalities in heart rate, PR interval, QRS interval, QTcF, QTcP, ΔQTcF, and ΔQTcP.

All evaluable local ECG data with clinically significant prolongation of the QTc interval (QTcP or QTcF ≥ 500 ms or ΔQTcP or ΔQTcF ≥ 60 ms) were reevaluated by certified cardiologists in a central laboratory. All ECG summary statistics were repeated for diphenhydramine premedication as a potential covariate in the analyses; patients had either received no premedication or ≥ 1 dose of diphenhydramine.

In addition, a random sample of patients enrolled in JAVELIN Solid Tumor and JAVELIN Merkel 200 with two available ECG measurements (including one at baseline) were randomly chosen to have their ECGs reevaluated by central read, irrespective of outlier status. All patients in the dose-escalation cohort of JAVELIN Solid Tumor also had their ECGs reevaluated by central read. These analyses were similar to those of a previous study that compared QTc measurements from digital ECG machines and a centralized core laboratory [24] and were performed to investigate the variability and quality of local reads compared with central reads.

### PK assessment and exposure–QTc analysis

In the JAVELIN Solid Tumor and JAVELIN Solid Tumor JPN dose-escalation cohorts, serial PK sampling was conducted, including sampling prior to and at the end of the first infusion and at 0.5, 1, 2, 4, 6, 12, 24, 36, and 48 h after infusion (24-, 36-, and 48-h samples were optional in JAVELIN Solid Tumor, and an additional sample was taken at 168 h after infusion in JAVELIN Solid Tumor JPN); in patients from the dose-escalation and dose-expansion cohorts, sparse PK sampling was conducted at trough and/or the end of infusion at multiple visits throughout the study. In JAVELIN Merkel 200, sparse PK sampling was conducted before infusion, at the end of infusion, and at 2 to 8 h after infusion at multiple visits throughout the study.

To assess the relationship between QTc data and avelumab, ECG time matched with avelumab concentrations was used. The exposure–QTc analysis included all patients who had ≥ 1 time-matched pair of PK and ECG measurements both before and within 2 h after infusion, and QTc data collected both in the presence and absence of diphenhydramine premedication were analyzed. For the exposure–QTc analysis, the following were assumed: more complex or advanced models beyond linear mixed-effects models were not needed to describe the data, any time difference ≤ 2 h between the observed QTc value and its paired avelumab serum concentration value did not affect results, parameters in the linear regression had a normal distribution for calculation of CIs, and the 3 studies had no differences that might affect the QT interval.

Four multivariable linear mixed-effects models were used to describe the quantitative relationship between serum concentration of avelumab and QTc or ΔQTc. The models used were based on the following equation: $Y_{ijk} = \beta_{0} + \beta_{1} C_{ijk} + \beta_{2} PM_{ijk} + \alpha_{0i} + \alpha_{1i} C_{ijk} + \epsilon_{ijk}$, where $Y_{ijk}$ denotes QTc or ΔQTc, *i* denotes patient, *j* denotes study day, *k* denotes nominal time (before infusion/2 h after infusion), $\beta_{0}$ denotes intercept, $\beta_{1}$ denotes slope of incidence of avelumab concentration (*C*), $\beta_{2}$ denotes premedication (PM) influence, $\alpha_{0i}$ denotes interpatient variability on the intercept, and $\alpha_{1i} C_{ijk}$ denotes interpatient variability on the slope. Models 1 and 3 were full models, which contained parameters to determine the effects of both avelumab and diphenhydramine premedication on QTc and ΔQTc, respectively. Models 2 and 4 were reduced models, which did not contain any parameters to measure the effect of diphenhydramine and, as such, only determined the relationship between avelumab and QTc and ΔQTc, respectively. The final models for QTc and ΔQTc data were selected based on the Akaike information criterion (AIC) and Bayesian information criterion (BIC).

## Results

### ECG summary analyses

Overall, 22,000 ECGs from 1818 patients, comprising 1681 patients from JAVELIN Solid Tumor, 51 from JAVELIN Solid Tumor JPN, and 86 from JAVELIN Merkel 200, were analyzed. Most patients analyzed had received the 10 mg/kg dose of avelumab (*n* = 1769); other doses received were 1 mg/kg (*n* = 4), 3 mg/kg (*n* = 18), and 20 mg/kg (*n* = 27).

Descriptive statistics of ECG parameters from patients in JAVELIN Solid Tumor who received the 10 mg/kg dose (*n* = 1643) were comparable to the parameters in the other studies and dose groups in this analysis. Up to week 49 of treatment in JAVELIN Solid Tumor (10 mg/kg avelumab), mean ΔQTcF and ΔQTcP were < 5 ms before infusion and approximately 5 ms at 2 h after infusion for all timepoints, which included ECGs from ≥ 100 patients, with a 90% CI upper bound of < 10 ms; ΔQTcF values were highly variable, with standard deviations (SDs) of approximately 20.0 ms (range 17.2–27.7 ms).

No clinically meaningful change in heart rate was observed at any dose level in the three studies (Table 1); no comparison between the three studies could be made due to the uncontrolled study settings. The use of diphenhydramine premedication did not have a clinically meaningful effect on the QT interval; QTcF > 500 ms was observed in 2.2% (30/1369 patients) with premedication and 3.1% (14/449 patients) without premedication.

**Table 1:** Frequency of patients from all studies with potentially clinically significant abnormalities during avelumab treatment at different doses based on locally read ECGs

Parameter	Patients *n* (%)				
	1 mg/kg (*n* = 4)	3 mg/kg (*n* = 18)	10 mg/kg (*n* = 1769)	20 mg/kg (*n* = 27)	Total (*N* = 1818)
Heart rate (bpm)					
≤ 50 and decrease from baseline ≥ 20	0 (0.0)	0 (0.0)	24 (1.4)	0 (0.0)	24 (1.3)
≥ 120 and increase from baseline ≥ 20	0 (0.0)	1 (5.6)	47 (2.7)	0 (0.0)	48 (2.6)
QRS interval (ms)					
≥ 120	0 (0.0)	1 (5.6)	165 (9.3)	4 (14.8)	170 (9.4)
PQ/PR interval (ms)					
≥ 220 and increase from baseline ≥ 20	0 (0.0)	0 (0.0)	109 (6.2)	4 (14.8)	113 (6.2)
QTcF interval (ms)					
> 450 and ≤ 480	0 (0.0)	4 (22.2)	312 (17.6)	7 (25.9)	323 (17.8)
> 480 and ≤ 500	0 (0.0)	0 (0.0)	63 (3.6)	2 (7.4)	65 (3.6)
> 500	0 (0.0)	0 (0.0)	44 (2.5)	0 (0.0)	44 (2.4)
ΔQTcF (ms)					
Increase from baseline > 30 and ≤ 60	0 (0.0)	7 (38.9)	393 (22.2)	6 (22.2)	406 (22.3)
Increase from baseline > 60	0 (0.0)	0 (0.0)	79 (4.5)	1 (3.7)	80 (4.4)
QTcP interval (ms)					
> 450 and ≤ 480	1 (25.0)	5 (27.8)	383 (21.7)	8 (29.6)	397 (21.8)
> 480 and ≤ 500	0 (0.0)	0 (0.0)	71 (4.0)	2 (7.4)	73 (4.0)
> 500	0 (0.0)	0 (0.0)	41 (2.3)	0 (0.0)	41 (2.3)
ΔQTcP (ms)					
Increase from baseline > 30 and ≤ 60	0 (0.0)	5 (27.8)	351 (19.8)	5 (18.5)	361 (19.9)
Increase from baseline > 60	0 (0.0)	0 (0.0)	65 (3.7)	2 (7.4)	67 (3.7)

*ECG* electrocardiogram, *QTcF* QTc derived from Fridericia’s formula, *QTcP* QTc derived from a project-specific formula

Based on locally read ECGs, 103 patients had on-treatment QTcF or QTcP ≥ 500 ms, or ΔQTcF or ΔQTcP ≥ 60 ms (Table 2). A total of 49 of 103 patients were evaluable for central read; patients were unevaluable due to deteriorated ECG paper or failure to send the ECG to the central laboratory before data cutoff. After centralized read, only 1 of 18 patients with QTcF > 500 ms and 5 of 43 patients with ΔQTcF or ΔQTcP > 60 ms were confirmed as outliers. All six patients with confirmed outlier ECGs had preexisting cardiac conditions [atrioventricular block (*n *= 1), bradycardia (*n* = 1), coronary artery bypass (*n* = 2), coronary artery disease (*n* = 1), hypercholesterolemia (*n* = 1), hypertension (*n* = 6), ischemic cardiomyopathy (*n* = 1), myocardial infarction (*n* = 1), pacemaker insertion (*n* = 2), or type 2 diabetes mellitus (*n* = 2)] or had been concomitantly treated with medication that has been associated with QT interval prolongation [dexchlorpheniramine (*n* = 1), diphenhydramine (*n* = 5), escitalopram (*n* = 1), loratadine (*n* = 1), ondansetron (*n* = 3), or oxycodone (*n* = 1)] [25–29].

**Table 2:** Overview of QTcF or QTcP outliers by local and central ECG read

Parameter	Patients, *n*		
	On-treatment outlier ECGs by local read	Evaluable ECGs for central read	On-treatment outlier ECGs confirmed by central re-read
QTcF or QTcP interval ≥ 500 ms per local machine–read ECG	45^b^	18^b^	1
ΔQTcF or ΔQTcP ≥ 60 ms per local machine^a^	87^a,b^	43^b^	5
Either condition fulfilled	103	49	6

*ECG* electrocardiogram, *QTcF* QTc derived from Fridericia’s formula, *QTcP* QTc derived from a project-specific formula

^a^Patients had evaluable baseline and on-treatment ECGs

^b^All evaluable outlier ECGs were centrally re-read

The random sample of patients enrolled in JAVELIN Solid Tumor and JAVELIN Merkel 200 (*n* = 180) provided 360 locally read ECGs that were randomly selected for reevaluation; 264 (73.3%) were evaluable for central read. Overestimation in locally read ECGs with QTcF < 400 ms was negligible with a mean of 0.8 ms; for ECGs with QTcF ≥ 400 ms, the mean overestimation was 10.3 ms (SDs of 14.4 and 15.3 ms, respectively). In the dose-escalation sample analysis (*n* = 53), locally read ECGs with QTcF < 400 and ≥ 400 ms were found to be overestimated in both short and long ranges, with mean differences of − 7.3 and − 13.9 ms (SDs of 9.4 and 12.0 ms), respectively.

### Exposure–QTc analysis

To assess the quantitative relationship between QTc and drug concentration, 2119 singlet locally read ECGs were analyzed from 670 patients across all three studies who had a baseline ECG measurement; ECGs were time matched with avelumab concentrations measured during PK assessments.

**Fig. 1:**
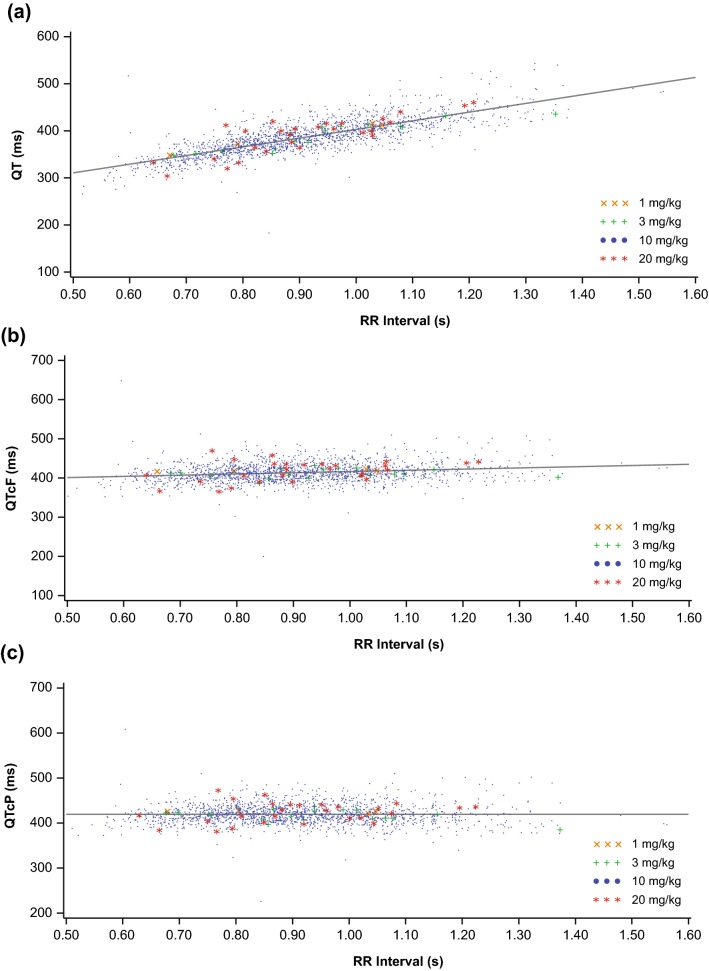
Regression of **a** baseline QT interval vs baseline RR interval (*n* = 1780, *P* < 0.0001^a^, *r* = 0.7912), **b** baseline QTcF interval vs baseline RR interval (*n* = 1780, *P* < 0.0001^a^, *r* = 0.1982), or **c** baseline QTcP interval vs baseline RR interval (*n* = 1780, *P *=0.9024^a^, *r* = − 0.0029). QTcF, QTc derived from Fridericia’s formula; QTcP, QTc derived from a project-specific formula. ^a^Refers to test of *r* = 0

Correlations between baseline QT, QTcP, and QTcF with the baseline RR interval were calculated (Fig. 1). As expected, a strong correlation was found between QT and RR (*r *= 0.7916). A weak but statistically significant correlation was found between QTcF and RR (*r *= 0.1941), whereas no correlation was found between QTcP and RR (*r *= − 0.0049). Therefore, QTcP was selected as the primary analysis variable; however, all analyses were also performed for QTcF.

Several multivariable linear mixed-effects models were developed for the exposure–QTc analysis, including full and reduced models that did or did not include a parameter to account for the effect of diphenhydramine (Table 3). For the analysis of QTcP and QTcF, the full model (Model 1) provided a better fit for the data per the AIC and BIC compared with the reduced model (Model 2); therefore, Model 1 was chosen as the final regression model. In Model 1, diphenhydramine induced a 3.9 ms increase in QTcP (90% CI, 2.4–5.5 ms; *P *< 0.001) and a 4.2 ms increase in QTcF (90% CI, 2.6–5.9 ms; *P *< 0.001). Avelumab concentration did not significantly affect QTcP or QTcF; when the diphenhydramine effect was accounted for, the serum concentration slope was 0.002 ms/(μg/mL) [90% CI, − 0.003–0.007 ms/(μg/mL); *P *= 0.512] for QTcP and 0.003 ms/(μg/mL) [90% CI, − 0.002–0.009 ms/(μg/mL); *P *= 0.310] for QTcF.

**Table 3:** Comparison of regression models for QTc absolute values and ΔQTc vs avelumab concentration including (full model; Models 1 and 3) or not including (reduced model; Models 2 and 4) a parameter for diphenhydramine premedication

Parameter	Model	Variable	Coefficient ± SE	*P* value	90% CI
QTcP	1	Serum concentration slope, ms/(μg/mL)	0.002 ± 0.003	0.512	− 0.003–0.007
		Premedication intercept (ms)	3.935 ± 0.935	< 0.001	2.395–5.475
	2	Serum concentration slope, ms/(μg/mL)	0.011 ± 0.002	< 0.001	0.008–0.015
QTcF	1	Serum concentration slope, ms/(μg/mL)	0.003 ± 0.003	0.310	− 0.002–0.009
		Premedication intercept (ms)	4.227 ± 0.993	< 0.001	2.592–5.861
	2	Serum concentration slope, ms/(μg/mL)	0.014 ± 0.002	< 0.001	0.010–0.018
ΔQTcP	3	Serum concentration slope, ms/(μg/mL)	0.003 ± 0.003	0.266	− 0.002–0.009
		Premedication intercept (ms)	3.363 ± 0.918	< 0.001	1.851–4.874
	4	Serum concentration slope, ms/(μg/mL)	0.012 ± 0.002	< 0.001	0.008–0.015
ΔQTcF	3	Serum concentration slope, ms/(μg/mL)	0.005 ± 0.003	0.123	0.000–0.010
		Premedication intercept (ms)	3.610 ± 0.968	< 0.001	2.017–5.204
	4	Serum concentration slope, ms/(μg/mL)	0.014 ± 0.002	< 0.001	0.010–0.018

*AIC* Akaike information criterion, *BIC* Bayesian information criterion, *QTcF* QTc derived from Fridericia’s formula, *QTcP* QTc derived from a project-specific formula

QTcP: AIC(full model) – AIC(reduced model) = − 19.2; ([BIC(full model) − BIC(reduced model)]/2) = − 9.6

QTcF: AIC(full model) − AIC(reduced model) = − 19.8; ([BIC(full model) − BIC(reduced model)]/2) = – 9.9

ΔQTcP: AIC(full model) − AIC(reduced model) = − 14.8; ([BIC(full model) − BIC(reduced model)]/2) = − 7.4

ΔQTcF: AIC(full model) − AIC(reduced model) = − 15.5; ([BIC(full model) − BIC(reduced model)]/2) = − 7.7

For the analysis of ΔQTcP and ΔQTcF, the full model (Model 3) also described the data more adequately than the reduced model (Model 4). In Model 3, diphenhydramine induced a 3.4 ms increase in ΔQTcP (90% CI, 1.9–4.9 ms; *P *< 0.001) and a 3.6 ms increase in ΔQTcF (90% CI, 2.0–5.2 ms; *P* < 0.001). Avelumab concentration did not have a statistically significant effect on ΔQTcP or ΔQTcF; the serum concentration slope was 0.003 ms/(μg/mL) [90% CI, − 0.002–0.009 ms/(μg/mL); *P *= 0.266] for ΔQTcP and 0.005 ms/(μg/mL) [90% CI, 0.0–0.01 ms/(μg/mL); *P* = 0.123] for ΔQTcF.

Across scheduled visits in JAVELIN Solid Tumor, the largest geometric mean maximum concentration (*C*_max_) values at 10 mg/kg (the approved-regimen dose) and 20 mg/kg (the highest dose tested) were 307 and 505 μg/mL, respectively. For patients who received diphenhydramine, the full model (Model 3) predicted a small increase in both ΔQTcP (3.5 and 4.2 ms for avelumab 10 and 20 mg/kg, respectively) and ΔQTcF (3.7 and 4.7 ms for avelumab 10 and 20 mg/kg, respectively), with a 90% CI upper bound of < 7 ms for both the 10 and 20 mg/kg doses (Table 4). For patients who did not receive diphenhydramine, ΔQTc at *C*_max_ would be even smaller.

**Table 4:** Model-predicted ΔQTcP and ΔQTcF at avelumab *C*_max_ in patients who received diphenhydramine in the 10 and 20 mg/kg dose cohorts of JAVELIN Solid Tumor

Parameter	Dose level	Observed maximum geometric mean *C*_max_ (μg/mL)^a^	Model-estimated ΔQTc at C_max_ (ms)	90% CI
ΔQTcP	10 mg/kg	307	3.492	(2.141–4.842)
	20 mg/kg	505	4.172	(2.066–6.279)
ΔQTcF	10 mg/kg	307	3.650	(2.214–5.087)
	20 mg/kg	505	4.650	(2.423–6.877)

*C*_max_ maximum serum concentration, *QTcF* QTc derived from Fridericia’s formula, *QTcP* QTc derived from a project-specific formula

^a^Geometric mean values are the largest observed concentrations at the end of infusion in JAVELIN Solid Tumor across all visits, where *n* > 3

## Discussion

The results of these analyses, performed in > 1800 patients with advanced solid tumors pooled from three studies, show that avelumab does not have any clinically relevant effect on cardiac repolarization. These analyses also show that avelumab coadministered with diphenhydramine, which is commonly given prior to avelumab treatment as prophylaxis for infusion-related reactions, does not have a clinically meaningful effect on the QTc interval.

In this analysis, we analyzed QTc values based on both Fridericia’s formula (QTcF) and a project-specific formula (QTcP) because QTcF did not completely remove the influence of RR on QTc. QTcP was chosen as the primary study endpoint of the exposure–QTc analysis due to its lack of correlation with RR. QTcF was also analyzed because it is commonly used in QTc analyses and, as such, was chosen as the secondary endpoint of the exposure–QTc analysis. Overall, results from the exposure–QTcF and QTcP analyses were consistent. All ECG analyses were repeated to test the effect of diphenhydramine on QTc prolongation, and the exposure–QTc analysis used linear mixed-effects models that included diphenhydramine as a covariant.

There were limitations associated with this study, including the lack of a controlled setting in which ECG measurements were taken. In the ECG summary analyses, there was a large degree of variation observed across different sites and countries, which made evaluation of the data challenging. Variability in QTcF absolute values resulted in an SD of approximately 20 ms for most timepoints. This variability could have been caused by concomitant medicines (e.g., antiemetics), known effects of diphenhydramine premedication [25], or underlying cardiovascular conditions; however, the analysis of this variation was not possible due to the lack of a placebo or control arm. Despite the aspects of the study design being suboptimal for exposure–QTc and ECG outlier analyses, the analysis objectives were met in accordance with ICH recommendations [19]: data were pooled from multiple studies that used the same methods of ECG measurement and analysis, and covered a wide range of avelumab doses; the exposure–QTc analysis used a robust modeling approach that included diphenhydramine premedication as a covariate; and locally read ECG data were reevaluated in a central laboratory, and the results of which, in line with reports from the previous studies [24], highlight the benefits of centrally read ECGs compared with locally read ECGs.

Only 6 of the 49 patients with locally read outlier ECGs evaluable for central read had confirmed outlier ECGs after reevaluation by central read (QTcF > 500 ms in one patient and ΔQTcF or ΔQTcP > 60 ms in five patients). All six patients had a history of cardiovascular conditions or were taking concomitant medications known to prolong the QT interval, which could explain the QTc findings.

To investigate the variability and quality of local reads compared with central reads, two additional ECG analyses were carried out: a random sample analysis of patients from JAVELIN Solid Tumor and JAVELIN Merkel 200 and a sample analysis of patients in the dose-escalation cohorts of JAVELIN Solid Tumor. Results from the random and dose-escalation sample analyses showed that overall QTcF was overestimated by locally read ECGs by a mean of 7.2 and 12.4 ms, respectively. In the dose-escalation sample analysis, overestimation occurred in both short and long ranges; however, in the random sample analysis, the overestimation of outliers in the short range was negligible. Based on these data, it was concluded that the frequency of QTc outliers in both short and long ranges was likely overestimated by local ECG reads. This is in line with previous studies, which have also reported potential overestimation of abnormalities in locally read ECGs compared with centralized reading [24].

In accordance with the concentration–QTc modeling approach recommended by the ICH E14 [19], we conducted an exposure–QTc analysis to describe the quantitative relationship between QTc and avelumab concentration. Diphenhydramine premedication, which is given prior to most avelumab infusions to reduce infusion-related reactions or for prophylactic purposes, has been reported to be associated with increased QTc prolongation and was, therefore, tested as a covariate in the exposure–QTc analysis. Multivariable regression models showed that diphenhydramine induced a small but significant effect on ΔQTc (ΔQTcP, 3.4 ms; ΔQTcF, 3.6 ms), consistent with the literature-reported risk of QTc prolongation [25]. The slopes of avelumab exposure vs ΔQTcP and ΔQTcF were not statistically different from zero after accounting for the effect of diphenhydramine [ΔQTcP: 0.003 ms/(μg/mL); *P* = 0.266; ΔQTcF: 0.005 ms/(μg/mL); *P* = 0.123]; these results showed that avelumab concentration had no statistically significant effect on the QT interval. Previous population PK analyses have suggested that diphenhydramine does not increase serum avelumab concentration (data on file) and, as such, the effect of diphenhydramine observed in this analysis is unlikely to be due to an effect on avelumab concentration.

For patients coadministered diphenhydramine, the model predicted a small increase in ΔQTc at the avelumab *C*_max_ for the 10 or 20 mg/kg dose, with a 90% CI upper bound of < 7 ms at both 10 and 20 mg/kg. This is below the threshold of concern (95% CI upper bound of 10 ms) [19] and, as such, these results suggest that avelumab coadministered with diphenhydramine does not cause QTc prolongation at the recommended therapeutic dose of 10 mg/kg or up to 20 mg/kg.

The absence of effect of avelumab on cardiac repolarization was anticipated because its large molecular size may prevent it from crossing plasma membranes and blocking hERG ion channels. Furthermore, the proposed mechanism of action of avelumab is not known to impact cardiac ion transport. These results are in line with QTc analyses of other immune checkpoint inhibitors, including nivolumab [30]. Furthermore, these findings are consistent with the nonclinical drug safety data for avelumab in cynomolgus monkeys, which showed that a high dose of avelumab (140 mg/kg administered every week for 13 weeks) did not cause any effect on cardiovascular function, proarrhythmic risk, or QT interval (data not shown). Finally, based on the data available at the time, an independent analysis carried out in 2016 by the US Food and Drug Administration also concluded that avelumab had no clinically relevant effect on QTc [31].

In conclusion, the clinical ECG analyses, including ECG summary and exposure–QTc analyses, presented here indicate that avelumab does not have any clinically relevant effect on cardiac repolarization.
